# Supplementary material for: Cytokine Signatures in Psoriatic Arthritis Patients Indicate Different Phenotypic Traits Comparing Responders and Non-Responders of IL-17A and TNFα Inhibitors
Source: Int J Mol Sci. 2023 Mar 28;24(7):6343. doi: 10.3390/ijms24076343 (PMC10093817; doi:10.3390/ijms24076343)
Supplement: Supplementary file 1 [file ijms-24-06343-s001.zip › Figure S1.pdf]

Figure S1: PCA correlation plot including biomarkers of PsA patients initiating MTX

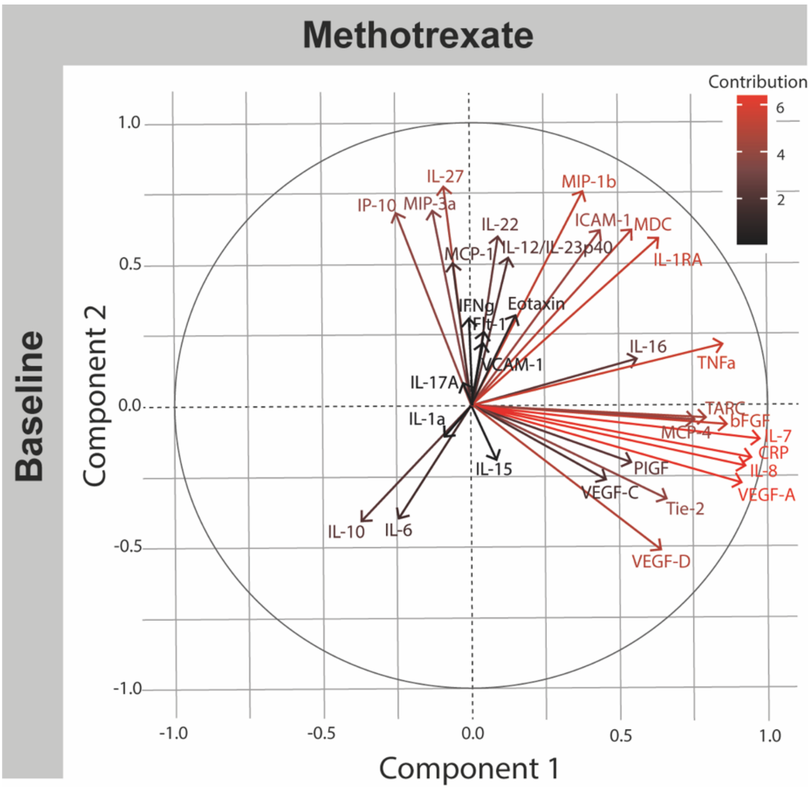

Correlations plot retrieved from the principal component analysis visualize the relationship between individual biomarker and the components. Component 1 and 2 uncorrelated described with the perpendicular axes from -1.0 to 1.0. The degree of contribution to the immune signature is illustrated with representing strong contribution to the component and black representing minor contribution. MTX; methotrexate, basic Fibroblast Growth Factor, Flt-1; Fms related Receptor Tyrosine Kinase-1, VEGFR1; Vascular Endothelial Growth Factor Receptor 1, PlGF; Placental Growth Factor, Tie-2; endothelial receptor tyrosine kinase, VEGF; Vascular Endothelial Growth Factor, IP-10; IFN-induced protein-10, CXCL; CXC chemokine ligand, MCP; monocyte chemoattractant protein, CCL; CC chemokine ligand, MDC; macrophage-derived chemokine, MIP; macrophage inflammatory protein, TARC; Thymus and activation regulated chemokine, IL; interleukin, IL-1RA; interleukin 1 receptor antagonist, IFN; interferon, TNF; Tumour Necrosis Factor, CRP; C-reactive protein, ICAM; Intercellular Adhesion Molecule, VCAM; Vascular Cell Adhesion Molecule.
