# Supplementary material for: Cytokine Signatures in Psoriatic Arthritis Patients Indicate Different Phenotypic Traits Comparing Responders and Non-Responders of IL-17A and TNFα Inhibitors
Source: Int J Mol Sci. 2023 Mar 28;24(7):6343. doi: 10.3390/ijms24076343 (PMC10093817; doi:10.3390/ijms24076343)
Supplement: Supplementary file 1 [file ijms-24-06343-s001.zip › Table S1.pdf]

Table S1: Biomarker level at baseline grouped by DAPSA50 responders and non-responders to treatment

	**TNFi responders (n=11)**	**TNFi non-responders (n=9)**	**p-value**	**IL-17i responders (n=7)**	**IL-17i non-responders (n=12)**	**p-value**
bFGF	311.94 (172.22-548.19)	331.07 (122.07-535.24)	0.824	420.41 (189.39-1363.28)	26.95 (128.25-415.00)	0.340
Flt-1/VEGFR1	1862.10 (1494.40-2206.90)	2271.0 (1725.0-2532.0)	0.295	1684.0 (1530.0-1807.0)	2101.0 (1765.0-2688.0)	0.100
PlGF	99.76 (85.84-104.65)	102.05 (90.84-110.34)	0.603	108.27 (105.26-120.62)	88.58 (52.88-122.68)	0.536
Tie-2	1.21x10^{5} (9.74.x10^{4}-1.34x10^{5})	1.28x10^{5} (9.10x10^{4}-1.31x10^{5})	0.882	1.12x10^{5} (7.81x10^{4}-1.22x10^{5})	1.18x10^{5} (8.19x10^{4}-1.45x10^{5})	0.536
VEGF-A	1837.0 (1698.0-2628.0)	2596.0 (1401.0-4016.0)	0.662	5679.0 (2368.0-6860.0)	2038.0 (1601.0-2945.0)	**0.005**
VEGF-C	1773.0 (1066.7-2113.1)	1936.0 (1545.8-2495.2)	0.503	3315.3 (2253.5-4489.8)	1250.5 (912.8-1637.5)	**0.022**
VEGF-D	2.25x10^{4} (1.94x10^{4}-2.82x10^{4})	2.00x10^{4} (1.61x10^{4}-2.56x10^{4})	0.503	2.66x10^{4} (1.93x10^{4}-3.11x10^{4})	2.12x10^{4} (1.51x10^{4}-2.54x10^{4})	0.340
Eotaxin	217.4 (147.3-378.4)	246.1 (231.4-418.0)	0.331	424.7 (314.3-545.1)	292.5 (214.8-427.3)	0.299
IP-10/CXCL10	576.40 (353.90-626.6.0)	428.4 (337.30-459.0)	0.412	655.3 (548.1.1014.6)	407.9 (314.8-602.5)	0.083
MCP-1/CCL2	122.18 (86.68-202.02)	126.01 (85.48-158.76)	0.824	257.60 (162.22-282.64)	122.20 (95.44-233.18)	0.261
MCP-4/CCL13	71.34 (51.79-130.59)	83.98 (70.58-170.03)	0.603	107.26 (74.19-141.13)	73.31 (63.72-100.79)	0.227
MDC/CCL22	1473.20 (761.0-1921.30)	1150.0 (1023.50-1756.1)	0.941	2039.6 (1828.5-2726.9)	1294.2 (1033.0-1784.2)	0.083
MIP-1β/CCL4	88.74 (65.38-104.59)	67.50 (45.47-107.35)	0.370	149.48 (128.15-216.46)	72.72 (61.28-98.67)	**0.002**
TARC/CCL17	140.85 (76.27-268-73)	143.84 (112.85-220.44)	1.000	310.0 (259.0-424.0)	122.01 (97.79-201.13)	**0.010**
IL-12/IL-23p40	99.76 (65.93-124.43)	80.10 (62.18-102.82)	0.710	199.47 (120.10-278.13)	111.60 (84.08-169.19)	0.227
IL-15	2.28 (1.95-2.53)	2.71 (2.16-3.02)	0.331	2.23 (2.02-2.83)	2.54 (2.180-2.87)	0.837
IL-16	279.1 (224.7-308.2)	247.00 (200.50-283.20)	0.503	289.7 (243.6-239.6)	231.73 (167.35-329.24)	0.299
IL-1 α	2.94 (1.76-4.79)	4.85 (2.80-5.96)	0.323	4.86 (1.85-8.17)	3.16 (2.18-5.08)	0.352
IL-7	3.84 (2.74-6.54)	4.71 (3.17-8.22)	0.656	4.85 (2.93-8.05)	2.77 (1.60-4.90)	**0.017**
IL-17A	4.64 (2.71-9.29)	4.85 (2.80-5.96)	0.094	11.23 (4.29-11.68)	4.74 (3.52-9.29)	0.966
IL-1RA	424.0 (22.3-520.6)	429.06 (396.72-566.72)	0.710	451.3 (427.7-1127.2)	550.9 (188.5-903.6)	0.482
IFNγ	4.73 (3.98-7.11)	5.65 (3.43-9.31)	0.941	4.58 (3.87-7.18)	6.75 (3.13-12.35)	0.536
IL-10	0.24 (0.17-0.42)	0.15 (0.14-0.23)	0.175	0.32 (0.22-0.39)	0.16 (0.14-0.22)	**0.045**
IL-6	2.28 (1.21-3.21)	1.12 (0.90-1.77)	0.230	1.67 (1.28-6.51)	1.28 (1.14-1.50)	0.120
IL-8	4.61 (4.09-5.18)	4.88 (4.58-5.77)	0.503	8.39-5.93-11.69)	4.34 (3.37-5.30)	**0.004**
TNFα	1.74 (1.33-2.00)	1.26 (2.217-4.22)	0.456	2.45 (1.83-36.25)	1.93 (1.43-2.36)	0.167
IL-22	1.31 (0.86-1.79)	1.70 (1.10-4.20)	0.331	1.323 (100-1.92)	1.95 (0.68-2.60)	0.711
IL-27	1002.90 (865.20-1745.80)	770.00 (542.00-1533.00)	0.370	1425.5 (1278.2-1591.1)	1140.4 (950.4-1359.2)	0.196
MIP-3α/CCL20	6.34 (5.26-10.73)	8.79 (5.76-12.23)	0.824	14.62 (12.28-22.41)	8.71 (5.68-10.98)	**0.036**
CRP	1.54x10^{7} (6.97x10^{8}-2.75x10^{8})	1.52x10^{7} (1.34x10^{7}-2.75x10^{8})	0.037	2.13x10^{8} (1.95x10^{7}-5.83x10^{8})	1.33x10^{8} (5.19x10^{7}-1.76x10^{8})	**0.009**
ICAM-1	7.86x10^{6} (6.80x10^{6}-8.25x10^{6})	7.29x10^{6} (6.23x10^{6}-7.87x10^{6})	0.447	8.72x10^{6} (7.84x10^{6}-1.05x10^{7})	8.25x10^{6} (7.03x10^{6}-9.58x10^{6})	0.592
VCAM-1	7.03x10^{6} (6.60x10^{6}-9.68x10^{6})	7.87x10^{6} (6.37x10^{6}-8.92x10^{6})	1.000	8.95x10^{6} (7.67x10^{6}-9.88x10^{6})	8.14x10^{6} (7.39x10^{6}-9.37x10^{6})	0.592

Biomarker levels at baseline were presented with medians and corresponding interquartile ranges. Response to treatment was defined by DAPSA50 (yes/no) corresponding to a 50% improvement in DAPSA from baseline to follow-up. DAPSA; Disease Activity in Psoriatic Arthritis, TNFi; Tumour Necrosis Factor-alpha inhibitor, IL-17i; Interleukin 17 inhibitor, bFGF; basic Fibroblast

Growth Factor, Flt-1; Fms related Receptor Tyrosine Kinase-1, VEGFR1; Vascular Endothelial Growth Factor Receptor 1, PlGF; Placental Growth Factor, Tie-2; endothelial receptor tyrosine kinase, VEGF; Vascular Endothelial Growth Factor, IP-10; IFN-induced protein-10, CXCL; CXC chemokine ligand, MCP; monocyte chemoattractant protein, CCL; CC chemokine ligand, MDC; macrophage-derived chemokine, MIP; macrophage inflammatory protein, TARC; Thymus and activation regulated chemokine, IL; interleukin, IL-1RA; interleukin 1 receptor antagonist, IFN; interferon, TNF; Tumour Necrosis Factor, CRP; C-reactive protein, ICAM; Intercellular Adhesion Molecule, VCAM; Vascular Cell Adhesion Molecule.
