# Supplementary material for: Cytokine Signatures in Psoriatic Arthritis Patients Indicate Different Phenotypic Traits Comparing Responders and Non-Responders of IL-17A and TNFα Inhibitors
Source: Int J Mol Sci. 2023 Mar 28;24(7):6343. doi: 10.3390/ijms24076343 (PMC10093817; doi:10.3390/ijms24076343)
Supplement: Supplementary file 1 [file ijms-24-06343-s001.zip › Table S2.pdf]

Table S2: Principal component analysis loadings of MTX initiators

	MTX initiators	
Biomarker	C1	C2
bFGF	**0.865**	-0.063
Flt-1	0.040	0.265
PlGF	**0.541**	-0.200
Tie-2	**0.661**	-0.327
VEGF-A	**0.915**	-0.270
VEGF-C	0.457	-0.260
VEGF-D	**0.641**	**-0.510**
Eotaxin	0.150	0.324
IP-10	-0.258	**0.684**
MCP-1	-0.065	**0.508**
MCP-4	**0.755**	-0.043
MDC	**0.541**	**0.629**
MIP-1β	0.374	**0.763**
TARC	**0.793**	-0.039
IL12/IL23p40	0.122	**0.528**
IL-15	0.084	-0.192
IL-16	**0.560**	0.171
IL-1α	-0.095	-0.190
IL-7	**0.975**	-0.113
IL-17A	-0.031	0.085
IL-1RA	**0.631**	**0.597**
IFNγ	-0.002	0.312
IL-10	-0.374	-0.408
IL-6	-0.249	-0.398
IL-8	**0.928**	-0.210
TNFα	**0.849**	0.224
IL-22	0.087	**0.604**
IL-27	-0.097	**0.781**
MIP-3α	-0.135	**0.694**
CRP	**0.946**	-0.179
ICAM-1	0.433	0.626
VCAM-1	0.038	0.223

Loadings or correlation coefficients for component 1 and component 2 of the principal component analysis in PsA patients initiating treatment with MTX. Grey markings represent biomarkers with >5% contribution to the component. Bold text represents significant loading factors >0.500. bFGF; basic Fibroblast Growth Factor, Flt-1; Fms related Receptor Tyrosine Kinase-1, VEGFR1; Vascular Endothelial Growth Factor Receptor 1, PlGF; Placental Growth Factor, Tie-2; endothelial receptor tyrosine kinase, VEGF; Vascular Endothelial Growth Factor, IP-10; IFN-induced protein-10, CXCL; CXC chemokine ligand, MCP; monocyte chemoattractant protein, CCL; CC chemokine ligand, MDC; macrophage-derived chemokine, MIP; macrophage inflammatory protein, TARC; Thymus and activation regulated chemokine, IL; interleukin, IL-1RA; interleukin 1 receptor antagonist, IFN; interferon, TNF; Tumour Necrosis Factor, CRP; C-reactive protein, ICAM; Intercellular Adhesion Molecule, VCAM; Vascular Cell Adhesion Molecule.
