# Supplementary material for: Cytokine Signatures in Psoriatic Arthritis Patients Indicate Different Phenotypic Traits Comparing Responders and Non-Responders of IL-17A and TNFα Inhibitors
Source: Int J Mol Sci. 2023 Mar 28;24(7):6343. doi: 10.3390/ijms24076343 (PMC10093817; doi:10.3390/ijms24076343)
Supplement: Supplementary file 1 [file ijms-24-06343-s001.zip › Table S3.pdf]

Table S3: Biomarkers evaluated with the Meso Scale Diagnostics (MSD) V-PLEX Human Biomarker 54-Plex

Kit	Biomarker
Angiogenesis Panel	VEGF-A†, VEGF-C, VEGF-D, Tie-2, sFlt-1, PIGF, bFGF
Cytokine Panel 1	GM-CSF, IL-1α, IL-5, IL-7, IL-12/IL-23p40, IL-15, IL-16, IL-17A†, TNFβ, VEGF-A†
Cytokine Panel 2	IL-17A/F, IL-17B, IL-17C, IL-17D, IL-1RA, IL-3, TSLP, IL-9
Chemokine Panel	Eotaxin, MIP-1β, Eotaxin-3, IL-8†, TARC, IP-10, MIP-1α, MCP-1, MDC, MCP-4
Th17 Panel	IL-21, IL-22, IL-23, IL-27, IL-31, IL-17A†, MIP-3α
Proinflammatory Panel	IFNγ, IL-1β, IL-2, IL-4, IL-6, IL-8†, IL-10, IL-12p70, IL-13, TNFα
Vascular Injury Panel	SAA, CRP, VCAM-1, ICAM-1

† Biomarker presence in two panels. IFN; interferon, IL; interleukin, TNF; Tumor Necrosis Factor, CCL; Chemokine (C-C motif) Ligand (chemokine subgroup 'CC'), CXCL; chemokine (C-X-C motif) Ligand, MIP; Macrophage Inflammatory Protein, TARC; Thymus- and Activation-Regulated Chemokine), IP; Interferon-gamma-indcued-Protein, MCP; Monocyte Chemoattractant Protein, GM-CSF; Granulocyte Macrophage Colony Stimulating Factor, VEGF; Vascular Endothelial Growth Factor, TSLP; Thymic Stromal Lymphopoietin, Tie; Tyrosine kinase, sFlT; soluble Fms-Like Tyrosine kinase, PlGF; Placental Growth Factor, bFGF; basic Fibroblast Growth Factor, SAA; Serum Amyloid A, CRP; C-Reactive Protein, VCAM; Vascular Cell Adhesion Molecule, ICAM; Intercellular Adhesion Molecule.
